# Self-Reported Energy Trajectories Predict Adverse Health Outcomes in Older Adults

**DOI:** 10.1093/geroni/igaa057.2880

**Published:** 2020-12-16

**Authors:** Briana Sprague, Xiaonan Zhu, Rebecca Ehrenkranz, Qu Tian, Theresa Gmelin, Nancy Glynn, Andrea Rosso, Caterina Rosano

**Affiliations:** 1 University of Pittsburgh, Pittsburgh, Pennsylvania, United States; 2 University of Pittsburgh Graduate School of Public Health, Pittsburgh, Pennsylvania, United States; 3 National Institute on Aging, Bethesda, Maryland, United States; 4 School of Public Health, University of Pittsburgh, Pittsburgh, Pennsylvania, United States

## Abstract

Declining energy may indicate homeostatic dysregulation and predict adverse health outcomes. We hypothesized that declining energy would predict greater frailty (1-10), greater mortality, and faster mood (CES-D) and cognition (3MS) decline over time. This observational cohort studies included 2,443 older adults (mean age=74.6, 62.5% White, 47.8% men) from the Health ABC Study with up to eight years of data. Energy was assessed using a single-item question about prior month’s energy (baseline mean=6.7, SD=1.7, range=0–10, lower=less energy). We used linear mixed models to create energy change scores (mean=-.07 points/year, SD=.05, range=-0.32-0.21, negative=decreased energy). In regression models adjusting for baseline outcome performance and energy and demographics, declining energy predicted greater frailty (β=-2.72, 95%CI = -3.39,-2.06), greater mortality (hazard ratio=.07, p<.001), and faster CES-D (β=-.93, 95%CI=-1.10,-0.75) but not 3MS decline. Energy changes are easy to assess and predict clinically-relevant outcomes. Future work should consider mechanisms of declining energy on disability-related outcomes. Part of a symposium sponsored by Brain Interest Group.

